# Integration of USDA Food Classification System and Food Composition Database for Image-Based Dietary Assessment among Individuals Using Insulin

**DOI:** 10.3390/nu15143183

**Published:** 2023-07-18

**Authors:** Luotao Lin, Jiangpeng He, Fengqing Zhu, Edward J. Delp, Heather A. Eicher-Miller

**Affiliations:** 1 Department of Nutrition Science, College of Health and Human Sciences, Purdue University, West Lafayette, IN 47907, USA; lin1199@purdue.edu; 2 School of Electrical and Computer Engineering, College of Engineering, Purdue University, West Lafayette, IN 47907, USA; he416@purdue.edu (J.H.); zhu0@purdue.edu (F.Z.); ace@ecn.purdue.edu (E.J.D.)

**Keywords:** food image database, food identification, nutrition assessment, database development, food category, food codes, nutrient composition

## Abstract

New imaging technologies to identify food can reduce the reporting burden of participants but heavily rely on the quality of the food image databases to which they are linked to accurately identify food images. The objective of this study was to develop methods to create a food image database based on the most commonly consumed U.S. foods and those contributing the most to energy. The objective included using a systematic classification structure for foods based on the standardized United States Department of Agriculture (USDA) What We Eat in America (WWEIA) food classification system that can ultimately be used to link food images to a nutrition composition database, the USDA Food and Nutrient Database for Dietary Studies (FNDDS). The food image database was built using images mined from the web that were fitted with bounding boxes, identified, annotated, and then organized according to classifications aligning with USDA WWEIA. The images were classified by food category and subcategory and then assigned a corresponding USDA food code within the USDA’s FNDDS in order to systematically organize the food images and facilitate a linkage to nutrient composition. The resulting food image database can be used in food identification and dietary assessment.

## 1. Introduction

Dietary assessments collect information on an individual’s food and beverage intake over a period of time that may be linked to a nutrition composition database and used to determine their intake of energy, nutrients, or other dietary components [1]. Dietary recalls, food frequency questionnaires, and food records/diaries are common methods utilized for dietary data collection. All methods place a significant burden of time and tedium on the participants and researchers who will consequently need to match each food reported to a similar food in a food composition database. Furthermore, the participant’s act of recording their diet may affect their intake (reactivity), as the participant may be less likely to eat foods they are asked to report [2]. In order to reduce the burden of recording and potentially reduce reactivity, new imaging technologies have been created to allow a participant to record foods and beverages by imaging the foods/beverages before intake with the camera of a cellphone, then imaging the absence of the food or beverage after their intake is complete. The before and after intake of these food/beverage images can then be processed to automate the identification of foods/beverages and their portion estimations [3,4,5,6,7,8]. These new technologies include deep learning-based methods and heavily rely on the quality of the available food image databases to accurately automate the identification of the foods/beverages. The images of foods and beverages comprising the food image database are already labeled, facilitating the linkage of the food/beverages in the participant’s image to a visually similar food/beverage in a food composition database. Therefore, the food image database is a critical component to accurately identify the food/beverage and match it to a similar item in a food composition database where the calories, nutrients, and other food composition can be derived per the amount of that food. With the development of mobile technology, a variety of image-based dietary assessment methods have been created [9]. The approaches have been shown to be well-received by participants due to a lower reporting burden, and their use may potentially replace conventional dietary assessment methods [10]. However, the accuracy of image-based identification and estimation is a concern with these methods, even though similar or better estimates have been shown compared to traditional dietary assessment [10], which also requires accurate self-reporting of dietary intake. An additional benefit of image-based identification could be a reduction in the researcher’s time and the financial burden of traditional dietary assessment. This study is the first to describe the creation of an image database and linkage to a nutrient database in order to derive nutrient compositions for image-based dietary assessment.

Despite the development of imaging technology to identify foods and beverages, food image databases that are transparently created, comprehensive, publicly available, and that link to federally maintained food composition databases do not currently exist [11,12,13,14,15,16,17,18,19]. Food image databases that do exist contain limited food types, are not focused on commonly consumed U.S. foods [11,14,15], are tailored to specific cultural dietary patterns (e.g., Chinese [20] and Japanese [12,21]), and are not linked to federally maintained food composition databases [9,10,11,12,13]. Another limitation of the currently available food image databases is the absence of bounding boxes in the images [22,23,24,25,26,27]. Bounding boxes separate different foods in the image and are important to mark the location of each food item for food identification. Furthermore, the identification and classification of the foods/beverages included in the images of the currently available food image databases are not transparently described nor is their process of inclusion in the image database described, such as how the list of foods to include was derived, how identification was assigned, and how errors were identified. Finally, current food image databases do not conform to any standard food categorization structure, leading to significant variation in how food items and images are classified in existing image databases. In addition to identifying specific food items, the food image databases could benefit from groupings of foods according to their food subcategories, categories, and broad food groups; for example, the item “pork rib” could be classified within the subcategory “pork”, which is within the category “meat”, and “meat” is within the food group “protein foods”. Taken together, these considerations suggest that the lack of categorization and organization of food items limits the ability of such image databases to be linked to a food composition database and, ultimately, to provide composition information on the dietary intake of participants.

Current national dietary data, national food composition databases, and food categorization structures provide the necessary data and framework upon which to create a comprehensive food image database focused on the most commonly consumed U.S. foods and beverages and those contributing most to energy intake and, therefore, the most important foods to capture in dietary assessment. The National Health and Nutrition Examination Survey (NHANES) is a nationally representative survey designed to collect information on the health and nutritional status of the U.S. population, where the most commonly consumed U.S. foods and those contributing most to energy intake can be derived via the dietary component of the NHANES, known as What We Eat in America (WWEIA). Foods and beverages reported in WWEIA are linked to the national food composition database, the United States Department of Agriculture (USDA) Food and Nutrient Database for Dietary Studies (FNDDS), via a USDA food code identifier [28], which contains not only single foods, but also mixed dishes, recipes, and ingredients. Once identified, food and beverage amounts can be estimated and converted into grams to determine portion sizes and nutrient compositions. The FNDDS is a highly comprehensive database that includes up-to-date nutrient and food composition information for thousands of foods and beverages that are commonly consumed in the U.S. and that are also culturally diverse, including all of the items reported by the participants of the NHANES. Information on a food item’s name, USDA food code, and description of the food can be obtained from the FNDDS. More specifically, the nutrient composition of the items within the FNDDS are based on laboratory analyses of food samples to ensure accuracy, and the database is regularly updated to reflect the changes in nutrient content and food supply. The nutrient compositions of the food in the FNDDS are listed per 100 g, allowing the nutrient compositions of the reported foods to be derived based on multiplying by the factor of their reported amount. Each food item within the FNDDS is assigned an 8-digit USDA food code. The USDA Food Surveys Research Group developed the classification structure for the FNDDS based on food categories utilized in WWEIA [29], and food items/food codes are grouped together accordingly in the FNDDS as a food subcategory, then food subcategories are grouped together as a food category based on usage and nutrient content. Food items/food codes are also updated with each iteration of the NHANES based on the foods reported, so some food codes are discontinued while others are added, all based on the foods reported as consumed in the new cycle of the NHANES.

This study describes the development of a food image database created collaboratively by a team of nutrition scientists and engineers using the nationally available data from the NHANES WWEIA and the corresponding database, the FNDDS. The population of focus for the development of this food image database was U.S. insulin takers. Little is known about the diets of individuals reporting taking insulin [30,31,32], but dietary intake is very important to diabetic patients using insulin because the dose of insulin depends directly on their dietary intake. Therefore, insulin takers provided an ideal target population for this study, as an example of tailoring an image database to the commonly consumed diet of a specific population of interest. This research is novel because it describes the first attempt to link food images to a national nutrition composition database in order to automate the estimation of the energy and nutrient composition of the imaged foods, which existing food image databases do not provide. The study objective was to develop methods to create a food image database that includes the most commonly consumed foods and those contributing the most to total energy intake as derived from the NHANES WWEIA data for U.S. insulin takers, which can be linked to the most recent version of a federally maintained national food composition database, the FNDDS (2017–2018), enabling the identification of food items, food subcategories, and food categories of WWEIA. That transparently describes the protocols for populating images as well as identifying and correcting errors that manifested during development.

## 2. Materials and Methods

### 2.1. Identify Commonly Consumed Food Types and Those Contributing the Most to Total Energy Intake in the Population of Interest

Due to constraints on time and resources, incorporating all possible foods consumed by U.S. insulin takers was not possible in this study, so the foods selected for inclusion were a prioritized optimization of the “most important” foods consumed by insulin takers that were the most frequently consumed and were those that contributed the most to total energy among U.S. adult insulin takers. These foods are likely to be the ones most frequently imaged by individuals and also the foods heavily contributing to energy and to the entire nutrient profile. A previous study identified these foods among adult U.S. insulin takers using the NHANES 2009–2016 [33] and used them to inform the selection of food types important for insulin takers that should be included in the food image database.

The next step was to consider how the selected foods could be structured to a systematic food classification system. The WWEIA food classification system was chosen, as this system aligns with the FNDDS food composition database to identify foods and derive their nutrition composition using a mobile telephone application designed to assess dietary intake, the Monitoring Eating and Lifestyle for Diabetes Management (MEAL-DM). The MEAL-DM is designed to provide an accurate account of daily food and nutrient intake. The application of the WWEIA system is ideal for broad applicability due to its role in the national U.S. nutrition monitoring systems and also because of its alignment with the food groups and dietary guidance of the Dietary Guidelines for Americans. The WWEIA food classification system is structured by specific food items, food subcategories, food categories, and most broadly, the food groups. The food groups in the WWEIA include protein foods, mixed dishes, grains, snacks and sweets, fruits, and vegetables. For the food image database designed for use with the MEAL-DM system, the term “food type” was used as the main level of categorization to distinguish it from the terms used in the WWEIA food classification system, as the food-type level of foods included a mix of some WWEIA food subcategories (e.g., melons) and other WWEIA food items (e.g., guacamole). The food-type level of foods was designed to ensure that foods similar in appearance and similar in nutrient composition were included in the same food type. Exact matches of WWEIA subcategories and items could not be made with the classification system levels because visual information is used in the MEAL-DM to identify foods, whereas the WWEIA organization relies on the foods and their relationships to similar foods and/or alternative preparations or ingredients that may not always be visually identified. Therefore, after assignment of food items to each food type, food types were assigned to a food group aligning with the WWEIA food groups. Once the categorization of each food item and food type was complete, each food type was assigned a general USDA food code corresponding to its specific WWEIA food subcategory. If a more specific USDA food code for each food item within the “food type” genre could be assigned, then the USDA food code was assigned at the “food item” level, in addition to the “food type” level.

Several issues arose in the classification of food types in the food image database. For example, some foods that were similar in nutrient content were not visually similar, such as a chicken breast when compared with a chicken thigh. If these two food items were classified as the same food type in the food image database under the food type “chicken, whole pieces” and grouped to a more generic WWEIA food category, it would be difficult to train the system to identify this food type because of the diversity of images within this food type. The use of such food types may result in poor classification performance. Therefore, this food type needed to be broken down to “chicken breast” as one food type and “chicken thigh” as another food type due to the visual dissimilarity. In addition, some food items that were visually similar may have very different nutrient composition, such as meatloaf and a loaf of bread. These two foods should be assigned to different food types in the food image database for accurate nutrient analysis. This situation represents a more difficult challenge compared with the items that are visually different but similar in nutrient composition. Such problems may require the mining of additional contextual information and remain as future opportunities for advancing the accuracy of food image databases. Yet, the issue presented by foods with similar nutrition compositions and different images (or similar images and different nutrition compositions) made salient the importance of choosing a primary classification structure for the food image database. So, the top 100 food types were prioritized from the list of the most frequently consumed foods and from those that contributed the most to total energy among insulin takers except for baby foods, condiments and sauces, sugars, fats, and oils. Next, each food type was evaluated further for inclusion in the following steps: 1. Beverages: Due to the difficulty in distinguishing among different types of beverages, the food types of beverages, alcoholic beverages, and water were excluded from the database for this project but remain an opportunity for future work; 2. Available Online Food Images: Necessary food images from online sources were not available for some of the food types, which limited the training of the system, and they were excluded; 3. Visual and Nutritional Similarity/Dissimilarity: Visual distinction was necessary to create unique food types; therefore, when visual distinction was present but nutritional similarity existed, the food types were split into multiple food types. The final list included 82 food types to develop the food image database based on the WWEIA food subcategories and the commonly consumed food names in existing databases [34] as identified and agreed upon by both the nutrition science and engineering teams of this project.

### 2.2. Mine Images from the Web

After the selection of the food types and their assignment to the categorization structure, the database needed to be populated with food images. For this task, at least 100 images were mined from the web for each of the 82 food types by the engineering team because at least 100 images were expected to cover the variety of settings, lightings, and other variations in images for each food type and to provide sufficient material to train the system databases [34]. A web crawler [35], or internet bot that automatically and systematically indexes the contents of a website or the internet, was used by the engineering team to search for and download a large set of food images from Google Images based on the food types identified in the previous step. Keywords of the food types were used to find the images through Google Images. The images included professionally photographed recipe pictures, dish photos that consumers took in real life, and so on. This process was challenging for several reasons: First, there were many “noisy” images that did not contain foods or that contained unreal foods, such as when “apple” was searched, the Apple Inc. logo image was captured, as well as sketches of food, images of plastic foods, and paintings of foods. To remove the “noisy” images, a Faster R-CNN [36] was trained for food region detection [35]. By using this tool, these “noisy” images were automatically removed, and images containing real foods were retained in the food image database. Second, choosing the key search terms was also challenging. For each food type, the original food name from WWEIA was used to search and identify food images. However, for some food types, only a limited number of images was found by using the original food names, so different key search words were needed to try to find more images. For example, “frankfurter sandwich” is a WWEIA food category name, but “hot dog” is a more common term for the food type, and therefore, “hot dog” was used in the search. Third, since there is no existing federal database for image analysis, subjective decisions were required in the process. For example, 100 images designated to a particular food type would be considered adequate based on the engineering team’s estimation of the expected performance for the identification and complexity of the deep learning model; however, this approach may not be standard across the research literature. This subjective decision was made given that a smaller number of images was not enough to train the system to identify food for automatic food recognition purposes. When less than 100 images were found, the food type was reevaluated and considered for combination with another food type or for removal from the image database. Eight food types in the creation of this food image database were removed or combined with other food types because of similarity or a lack of sufficient images identified in the mining process. For example, “cake” and “cupcakes” were combined as one food type. Noisy images such as sketches of food were also removed as part of this process by the engineering team using an online crowdsourcing tool [35]. After this step, 16,114 images in 74 food types were included in the food image database.

### 2.3. Annotate the Food Images

The next step was to identify the foods in the food images collected. The bounding box, defined as the box drawn around the single food item in an image, was created along with the food name for each single food item by the engineering team. Bounding boxes can be drawn efficiently through the web annotation tool [35] by clicking and dragging using a computer mouse on the web interface. Each food image was reviewed by a human annotator to verify that the image was a food, assign the bounding box, and remove all images that were not foods or where the food type was not clear. One bounding box identified only one food; if the image contained multiple foods, the image was duplicated and assigned to the different food types within the food image database, then different bounding boxes were placed on the different foods of respective specific food types. For each bounding box, researchers from the engineering team manually assigned a food name to identify the food. After completing this step, the food image database included 74 food types, and 22,423 bounding boxes were marked on 16,299 food images.

### 2.4. Identify Range of Error for the Total Image Database

Next, the nutrition science team reviewed all the images in the database and identified the food images that were categorized into incorrect food types or where the bounding box was not placed on the correct food. The percentage of food images classified to food types that were incorrectly labeled and with incorrect box placements was assessed by tallying the number of incorrect images and then dividing by the total images in that food type (incorrect image % = (incorrectly labeled images + incorrect box placements)/total images of the food type). In addition to evaluating total database error, errors in the human image checking results of the mined images, the assigned bounding boxes, and the assignment of food categories in the food image database were also separately evaluated to determine if certain food groups or other areas were prone to errors using the study protocols. In addition, during the review in the previous step, researchers from the nutrition science team additionally checked for errors in food names that were added to food images in Section 2.3.

### 2.5. Review Food Images in the Food Image Database

Four reviewers from the nutrition science team were divided into two groups to independently review all of the mined images, bounding boxes, and food categories as well as to assign individual, specific FNDDS food item codes to identify each food image. The FNDDS food codes assigned by each independent reviewer were then compared to determine inconsistency. When different, independent reviewers functioned as tiebreakers to assign the final food code. The purpose of the two independent reviews of the nutrition science team was to check the previous work, correct the errors, decrease subjective errors in the final image database, and link the food image database to the FNDDS.

Errors were annotated from the two independent reviews, and recommendations to best resolve the checking results were made. In the first independent review, researchers suggested removing some images from the database due to (1) the foods in the images not being identifiable (food is too small or image is too vague), (2) the presence of raw foods in the images that would not be suitable for direct consumption such as raw ribs, and (3) duplicate images. Researchers also suggested moving some images to another food type because they were categorized incorrectly. Moreover, researchers also made suggestions for bounding box placement because some bounding boxes (1) were not placed on the correct food, (2) did not include all the food in the image, or (3) were only placed on a certain item in the image whereas the FNDDS food code assigned was relevant to the whole dish.

In the independent reviews, the nutrition science team also assigned a FNDDS food code to each food image. In certain cases, the image may have encompassed several FNDDS food codes, but ultimately, the nutrient composition information needed to be derived from one of the food codes. Based on previous work [33], the most frequently consumed food code was assigned as the overall food code so as to assign the food item with the highest probability of being consumed among those individuals using insulin [33]. Alternatively, a general FNDDS food subcategory code as “Not further specified” (“NFS”) may have also been assigned to food types in the food image database if the most frequently consumed food code did not capture most food items included in that type. For example, the most frequently consumed bean is pinto beans, yet pinto bean was not assigned as the general food code for the food type of beans because pinto bean does not capture the variation in the food type of beans, so “Bean, NFS” was assigned. One additional caveat was that previous work [33] used NHANES 2009–2016, and this study and the image database created here used FNDDS 2017–2018. This resulted in some of the most common food codes identified in the previous work being outdated, and in these cases, the most similar food codes to the outdated food codes in the FNDDS 2017–2018 database were assigned to the images.

Then, FNDDS food codes and WWEIA food categories [29] were used as the guide to further specify the food codes assigned in each image when further specification was possible. Each individual image of different food types in the food image database was reviewed to determine if the food in the image corresponded with a different or more specific food code. According to the images, alternative FNDDS food codes could be assigned if more specific food codes were available and appropriate within the FNDDS database. Food in some images may not have more specific food codes to be assigned, or, if the visual appearance was ambiguous and further specification was not possible, then a general food code that contains similar macronutrient distributions or a general food code that contains “NFS” or “NS (not specified)” was assigned. Sometimes, the general food code and the most commonly consumed food codes had different names but the same macronutrient content. This is because the NFS description takes into consideration the frequency of consumption of the more specific food codes [37]. Nutrient composition was also considered in the assignments, especially when the more specific food code may have very different nutrient composition compared to the general food code for that food item. For example, chocolate-coated strawberries have more calories, fat, and sugar content compared to the assigned food item code of “raw strawberries”, so “milk chocolate candy, with fruit and nuts” was assigned to the image of chocolate-coated strawberries.

After finishing the first independent review, the decision to retain duplicate images for image training purposes was made. The second independent review was conducted similarly by two different reviewers on the nutrition science team. Each reviewer independently reviewed the images in the database and determined the appropriate food code to assign and made suggestions to correct errors that were discovered. During the review, the reviewers also took notes on images, so their thoughts, justification for their decisions, and the details of the images were recorded for checking in the future. After the two independent reviews, 9431 out of 16,299 images had different food codes. Then, two additional reviewers in the nutrition science team reviewed all of these images that had discrepancies in the food code assignment from the two reviews and made the final decisions based on the prior assignments from the two independent reviews and their own judgement. All the reviewers were trained on the systematic classification and categorization of foods using WWEIA and the USDA food coding system before performing the assignments. Each image in the food image database was reviewed and evaluated at least twice, and about 58% of the images were checked three times. The engineering team then worked to update all food types and images in the database to produce the final food image database, which included 74 food types, and 22,061 bounding boxes were marked on 15,947 food images.

## 3. Results

### 3.1. Range of Errors in the Food Types by Food Group in the Food Image Database

The following section, corresponding to Table 1, describes results of the range of errors in food groups from populating the food image database with images using the online web crawler and the bounding box placement as described in Section 2.4.

Through the process of identifying the range of errors within the food image database, 6.42% of the total images mined online were categorized into incorrect food types, and 0.37% of the images had incorrect bounding box placement. When evaluated by food group, mixed dishes had the largest percent error of foods categorized into an incorrect food type, and protein foods had the largest percent error of images that had incorrect bounding box placement by humans.

### 3.2. Errors Identified in the First Two Independent Reviews of Food Image Database

The two independent reviews described in Section 2.5 identified different errors for each food type in the food image database, and examples are shown below in Table 2.

After the first review, 1523 images had problematic annotations: 1157 images were suggested to be removed because they were either images of raw foods that individuals do not usually eat raw, foods that were not able to be identified in the images, or duplicate images; 305 images were suggested to be moved to another food category; and 61 images were suggested to have their bounding boxes corrected. After the second review, 567 images had problematic annotations: 418 images were suggested to be removed and 149 images were suggested to have their bounding boxes corrected. 

### 3.3. Finalize the Food Images Included and Their Classification to Food Type within the MEAL-DM Food Image Database

As mentioned in Section 2.5, after cleaning the food image database by removing the images that did not meet the requirements above, the images were reassigned as the corrected food types and/or the placement of the bounding boxes was corrected. Table 3 shows two examples of food images that were assigned different food codes from the first two independent reviews and the final food code. 

In total, 15,947 images in 74 food types were finalized in the food image database. Examples are shown in Table 4. The 74 food types listed alphabetically included Almonds, Apple, Applesauce, Avocado, Bacon, Bagel, Baked potato, Bananas, Beans, Biscuit, Blueberries, Boiled egg, Breaded fish, Broccoli, Brownie, Burger, Burrito, Cabbage, Cake or cupcakes, Carrot, Chicken breast, Chicken nugget, Chicken tender, Chicken thigh, Chicken wing, Cinnamon bun, Coleslaw, Cookies, Corn, Croissant, Doughnut, Frankfurter sandwich, French fries, French toast, Fried egg, Fried rice, Green beans, Grilled salmon, Guacamole, Hash browns, Ice cream, Lasagna, Macaroni or noodles with cheese, Mashed potatoes, Meat loaf, Melons, Muffins, Nachos, Omelet, Pancake, Pasta mixed dishes, Pies, Pizza, Pork chop, Pork rib, Quesadilla, Quick bread, Sandwich, Shrimp, Soup, Steak, Stewed beef, Strawberries, Sushi, Taco or tostada, Tomatoes, Tortilla and corn chips, Tortillas, Tuna salad, Waffles, White and brown rice, Whole chicken, Yeast bread, and Yogurt. The complete FNDDS database contains values for additional nutrients including Water, Energy, Protein, Fat, Carbohydrate, Sugars, Fiber, Alcohol, Cholesterol, Saturated fatty acids, Monounsaturated fatty acids, Polyunsaturated fatty acids, Calcium, Copper, Iron, Magnesium, Phosphorus, Potassium, Selenium, Sodium, Zinc, Vitamin A, Vitamin C, Choline, Vitamin B-12, Vitamin E, Vitamin D, Folate, Vitamin K, Niacin, Retinol, Riboflavin, Thiamin, Carotene, Cryptoxanthin, Lutein + Zeaxanthin, Lycopene, Caffeine, and Theobromine.

In this study, methods to develop a food image database were devised and refined, and a food image database was created to facilitate the identification of foods and to link food images to their composition in a federal nutrient composition database. The resulting food image database contained 15,947 images of 74 food types. A few previous studies describe the creation of food image databases [11,12,13,14,15,16,17,18,19,20,21], but they only include limited food types, are not linked to federally maintained food composition databases, and did not provide descriptions of how the databases were created. The methods described here uniquely create the first food image database aligning to the WWEIA system of food classification, with USDA food codes assigned to each image to facilitate FNDDS linkage to derive the nutrition compositions for further nutrient analysis. Bounding boxes were placed on each food in the images to specify the items identified, separate different foods for downstream tasks, and provide the location of each food item for food identification. This newly created food image database included food types based on federal food classification that allow translation to federal nutrition guidance in the Dietary Guidelines for Americans and provide consistent food composition values including energy and nutrients according to national food composition databases that link to former research and national guidelines in contrast with prior food image databases lacking such information.

This food image database bridges the gap between the image reporting of foods and deriving food composition from a federally maintained nutrient database as well as facilitates the application of advanced algorithms for image analysis. For instance, existing identification methods merely determine food types without yielding nutritional content information. This limitation necessitates additional manual input to derive the necessary data for dietary assessment. By utilizing this food image database to train convolutional neural networks, it is possible to directly predict nutritional contents or food codes from input images. This advancement enables a truly automatic dietary assessment process. One of the novelties of the database is the consideration of visual, food group-based, and nutrition information in the structure and organization and even in the assignment of foods to the various levels (group, category, subcategory, or item). Furthermore, the most commonly consumed foods and those contributing the most to energy were used as a basis for the inclusion of the various food types and in the consideration of food code assignment to tailor the database to dietary assessment of a specific population group, adult insulin takers. The database linkage to the FNDDS using USDA food codes represents a major strength as it provides consistency with standard federal nutrition surveys, allowing the calculation of an array of macronutrients, micronutrients, energy, and other relevant food components of interest in dietary assessment. In addition, because of the diverse and extensive population of images, this food image database can facilitate food image identification even for low-quality images or those with insufficient lighting conditions. Assignment of the food label on the food images provides oversight on image mining and enables a more accurate prediction compared with other databases with automated identification or mined food identification. The detailed methods and transparent protocols using previous scientific evidence of the target population described in the creation of this database improves on existing descriptions of methods of creating food image databases.

Perfect and comprehensive identification of all foods may be difficult to achieve, and errors may still be inherent to food identification using this database. However, the detailed methods and errors discovered and documented here may improve the identification of bias and steps to improve on these methods in the future. For example, during the development of the food image database, errors were identified: 6.42% of the total images were categorized as incorrect food types, and 0.37% of the images had incorrect bounding box placement in Section 2.4. Overall, the mean error percentages of different food groups were low. High error percentages occurred the most in grains (brown rice, muffin, French toast) and mixed dishes (cheeseburger) compared to other food groups, which may be due to multiple reasons, including the similar appearance (muffin and cupcakes), complex composition (image of cheeseburger without cheese), or overlap with other groups (brown rice and other rice varieties, French toast and regular toast). In addition, most of the errors found in Section 3.1 were due to foods categorized into incorrect food types, which may have occurred when the engineering team assigned images into different food types prior to the incorporation of the food classification structure. In addition to evaluating the total database error, the errors due to the human image checking results of mined images, assignment of bounding boxes, and food types in the food image database differed by food groups. For example, the food group milk and dairy had the highest mean percentage of error for incorrectly categorized foods out of all of the food groups, as shown in Section 3.1. However, this group had fewer images compared to other food groups. Therefore, even though the error percentage of this group was comparatively higher, there were only 22 images with errors. Errors are critical to quantify to better understand the resulting determination of macronutrients that may be relevant in using the food image database to analyze the diets of insulin takers, the target group for application of this database. For example, foods such as grains containing carbohydrates are relevant to the management of diabetes. The classification of foods such as grains into the correct food types to obtain accurate nutrient information is important to determine carbohydrate intake and to help inform insulin dosing. If an item such as a cake was classified as a muffin, the error may make a difference in nutrient composition. Understanding the image errors (Section 2.4) associated with these groups (due to their importance in terms of insulin dosing and diabetes management), the extent of the error, type of error, and how the errors can be improved/eradicated is impactful to decision making in sequential steps (Section 2.5) of the process described here. Due to the errors found in Section 2.4, it was possible that more images had errors; therefore, the team decided to reevaluate all the images in the database.

This study also had limitations to be acknowledged. First, in the independent review process by the nutrition science team in Section 2.5, food codes were assigned, but more than 58% of the total images had different food codes between the two independent reviews. This kind of error underlies the challenges of food identification, even for humans. One reason is that certain food types may be hard to define. For example, a cheese sandwich could be any sandwich that contains cheese (including cheeseburgers), or a sandwich that includes only cheese (i.e., a typical “grilled cheese”). Fried rice may be any rice that appears to have been fried, or perhaps the assignment should only be for fried rice dishes that are culturally Asian. Reviewers may have struggled to assign foods such as a “biscuit” as a savory American biscuit (like those in biscuits and gravy), or as a cookie similar to a British biscuit. A second difficulty was the two-dimensional visual representation of three-dimensional foods where textural and context information is lost. Food can be visually very similar, such as yogurt, whipped cream, sour cream, assorted dips, and smoothies, but have other differing characteristics that may not allow distinguishability from the visual information alone. Sometimes, the food can be identified based on the context; for example, sour cream may be a probable classification when it is on the top of a baked potato. Yet, such information is not consistently available. Those assigning the classification may have to rely on their own judgement to make a subjective guess and choose the most accurate food code from the more than 7000 food codes of the FNDDS database. In addition, independent reviewers may have different standards of annotation through the process. In the first reviews, researchers did not assign food codes to the images that did not belong to that specific food type or to duplicates because those images were planned to be deleted. However, due to the purpose of training, in the second review, researchers decided to keep the duplicate images. The duplicate images were then assigned food codes in the second review, which caused differences between the two independent reviews. Furthermore, there were several challenges that became apparent in assigning food codes to the images because (1) the quality of some images was low, (2) lighting conditions were insufficient, (3) the food was covered by sauce, or (4) the ingredients or cooking methods of the foods in the images were not a perfect match to the food codes in FNDDS. Due to those challenges, the classification and linking of food codes remained difficult. Yet, these challenges highlight the contributions of this paper to food identification for the scientific community, as the challenges and methods to classify the food into different food categories and link the food images with food codes are documented for improvements by future investigators. The creation of this food image database represents a first attempt to build and improve on the methods and documentation of prior almost non-existent methodology. Second, the database includes a limited number, that is, 74 food types that do not represent all of the food included in the FNDDS nor all the foods that Americans consume such as beverages and salads. Food types such as beverages were excluded from the food image database because solid-food identification was prioritized due to the visual limitations of opaque containers in which beverages are often contained. In addition, many drinks and beverages are visually similar, such as “cola” and “diet cola” or “whole milk” and “fat free milk”. Some of these beverages, as in the example of cola and diet cola and the varying percentages of fat in milk, have the same visual appearance but different nutrition contents. In such cases, additional queries to the user may need to be introduced to collect this information that is not obtainable from the images alone. Beverages represent a potentially more challenging food type for visual identification compared with solid foods. Moreover, too many different beverage food types could complicate the development of the food image database, but in the future, they should be added into the database as an important source of nutrients. Third, another limitation is that the food codes that were assigned to the images may not be accurate due to a variety of reasons, such as the food in the images being difficult to identify. In this case, general food codes that were not further specified or were more commonly consumed may be assigned even though they may not be the most specific. A more general food code or the most frequently consumed food code within that food type may be a better match compared to alternative food code assignments. Fourth, during the process, the researchers used their own judgment to decide which food codes should be assigned to the images, which was subjective. The process of the two independent reviews and independent tiebreakers was a strength and helped to mitigate subjectivity in the final food code assignment. Fifth, the provision of other information such as the glycemic index may be a future addition to the system to help patients better understand their diets and to control their postprandial blood sugar level. Sixth, this food image database is based on the diet of American adults, so it may be difficult to apply to individuals in other countries; however, using this method of developing a food image database, other countries can create food image databases that link to their own nutrient composition databases.

In the future, food types such as salad and others that were not included in the current food image database should be added to include these additional commonly consumed foods for a more comprehensive and accurate food image database. The development and process of this food image database represents an example that may be applied to develop similar tailored food image databases for other special populations of interest. This database will be validated in a future pilot study where participants will use an image-based mobile application to record food and beverage intake, and the system will identify the foods that participants consume and compare them with the participants’ self-reported dietary recall. This food image database can also be used for dietary assessment and dietary identification training, among other useful applications.

## 4. Conclusions

This study describes the development of methods to create a food image database that is based on the USDA WWEIA food classification system and USDA FNDDS food composition database for food identification and nutrition analysis for use with image-based dietary assessment. This first-time link between a food image database and a national nutrition composition database described in this study holds promises for future dietary assessment. In the future, additional food items and types may be added to comprehensively include more foods that people consume and increase the accuracy of the food labels of the images in the database.

## Figures and Tables

**Table 1:** Range of errors of the MEAL-DM categorized food types by WWEIA food groups for images within the food image database.

Food Group (Number of Food Types)	Total Number of Images	Range of Error % for Food Types in Food Groups		Mean Error % for Food Groups	
		Wrong Food	Incorrect Box Placement	Wrong Food	Incorrect Box Placement
Milk and Dairy (1)	173	12.7%	0.0%	12.7%	0.0%
Grains (10)	2056	0–24.3%	0–1.6%	9.9%	0.3%
Mixed Dishes (14)	3165	0–24.5%	0–0.5%	8.1%	0.2%
Snacks and Sweets (10)	2555	0–14.8%	0–1.0%	5.3%	0.2%
Vegetables (12)	2664	0.8–19.2%	0–2.7%	5.1%	0.5%
Protein Foods (21)	4371	0–16.5%	0–2.8%	4.5%	0.7%
Fruits (6)	1315	0.5–13.0%	0–2.1%	3.2%	0.7%
Total (74)	16,299	0–24.5%	0–2.8%	6.4%	0.4%

**Table 2:** Examples of problem images in the two independent reviews of the MEAL-DM food image database with descriptions of the errors and suggested revisions.

Images	Errors	Revision
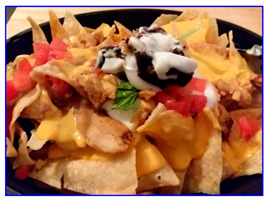	Incorrect food type (identified as taco)	Move to the food type of “Tortilla and corn chips”
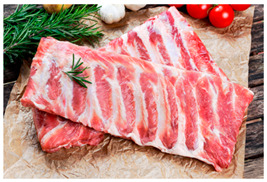	Raw food	Delete
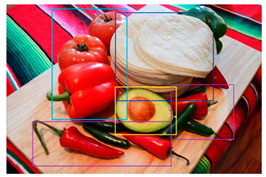	Incorrect bounding box (blue bounding box on the left was incorrectly placed on both the red bell pepper and tomato)	Two bounding boxes should be used to designate the red bell pepper and tomato separately
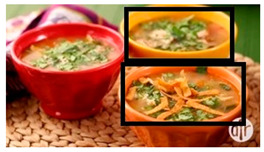	Incorrect bounding box (missing one bounding box for the soup on the left)	Add a bounding box to identify the soup on the left side of the image

**Table 3:** Examples of different food codes assigned to the same food image, the final food code assignment, and the reason for the assignment.

Images Number	Image	FNDDS Code 1 ^1^	Food Description 1 ^1^	FNDDS Code 2 ^2^	Food Description 2 ^2^	Final Code	Final Description	Notes ^3^
767361.jpg	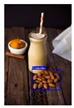	42100100	Almonds, NFS	42101130	Almonds, unsalted	42101130	Almonds, unsalted	Based on the image, almonds are not salted, so 42101130 Almonds, unsalted is a more suited food code for this image
754266.jpg	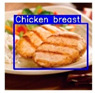	24120120	Chicken breast, NS as to cooking method, skin not eaten	24103050	Chicken, NS as to part, grilled without sauce, NS as to skin eaten	24123301	Chicken breast, grilled without sauce, skin not eaten	The final code specified the cooking method and excluded the skin that did not show in the image

^1^ FNDDS codes and food descriptions were assigned from the first round of review and to link food images to the FNDDS nutrition database. ^2^ FNDDS codes and food descriptions were assigned from the second round of review to link food images to the FNDDS nutrition database. ^3^ Notes were the reasons for selecting the final codes and descriptions.

**Table 4:** Examples of the food image database with nutrient information including 65 nutrients ^1^.

Image Number	Image	Food Broad Group (WWEIA)	Food Category (WWEIA)	Food Subcategory (WWEIA)	USDA Food Code	USDA Food Code Description	Standard Serving Size ^2^	Energy (kcal)	Protein (g)	Fat (g)	Carbohydrate (g)	Calcium (mg)	Vitamin D (mcg)
732381.jpg	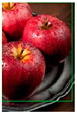	Fruits	Apple	Apple	63101000	Apple, raw	100 g	61	0.17	0.15	14.8	5	0
733566.jpg	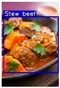	Mixed Dishes	Meat mixed dishes	Stew beef	27311310	Beef stew with potatoes and vegetables including carrots, broccoli, and/or dark-green leafy; tomato-based sauce	100 g	90	5.24	3.98	8.25	14	0
783254.jpg	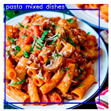	Mixed dishes	Pasta mixed dishes	Pasta	58146301	Pasta with tomato-based sauce and added vegetables, restaurant	100 g	158	3.47	7.85	18.13	14	0

^1^ Only select nutrients are shown from the complete food image database which includes Water (g); Energy (kcal); Protein (g); Fat, total (g); Carbohydrate (g); Sugars, total (g); Fiber, total dietary (g); Alcohol (g); Cholesterol (mg); Saturated fatty acids, total (g); 4:0 (g); 6:0 (g); 8:0 (g); 10:0 (g); 12:0 (g); 14:0 (g); 16:0 (g); 18:0 (g); Monounsaturated fatty acids, total (g); 16:1 (g); 18:1 (g); 20:1 (g); 22:1 (g); Polyunsaturated fatty acids, total (g); 18:2 (g); 18:3 (g); 18:4 (g); 20:4 (g); 20:5 n-3 (g); 22:5 n-3 (g); 22:6 n-3 (g); Calcium (mg); Copper (mg); Iron (mg); Magnesium (mg); Phosphorus (mg); Potassium (mg); Selenium (mcg); Sodium (mg); Zinc (mg); Vitamin A, RAE (mcg); Vitamin C (mg); Vitamin B-6 (mg); Choline, total (mg); Vitamin B-12 (mcg); Vitamin B-12, added (mcg); Vitamin E, alpha tocopherol (mg); Vitamin E, added (mg); Vitamin D (D2 + D3)(mcg); Folate, DFE (mcg); Folate, food (mcg); Folate, total (mcg); Folic acid (mcg); Vitamin K (mcg); Niacin (mg); Retinol (mcg); Riboflavin (mg); Thiamin (mg); Carotene, beta (mcg); Carotene, alpha (mcg); Cryptoxanthin, beta (mcg); Lutein + Zeaxanthin (mcg); Lycopene (mcg); Caffeine (mg); and Theobromine (mg). ^2^ Standard Serving Size: Quantity (whole number, fraction, or decimal) of a measure description to obtain the desired nutrient intake, which can be calculated by multiplying number of servings times the weight.

## Data Availability

The database described in the manuscript is made publicly and freely available without restriction at https://lorenz.ecn.purdue.edu/~vfn/ (accessed on 11 August 2022).
